# Electrostatic MEMS Two-Dimensional Scanning Micromirrors Integrated with Piezoresistive Sensors

**DOI:** 10.3390/mi15121421

**Published:** 2024-11-26

**Authors:** Yameng Shan, Lei Qian, Kaixuan He, Bo Chen, Kewei Wang, Wenchao Li, Wenjiang Shen

**Affiliations:** 1 School of Nano-Tech and Nano-Bionics, University of Science and Technology of China, Hefei 230026, China; ymshan2018@sinano.ac.cn; 2 Suzhou Institute of Nano-Tech and Nano-Bionics, Chinese Academy of Sciences, Suzhou 215123, China; 3 East China Institute of Photo-Electron IC, Bengbu 233000, China

**Keywords:** MEMS micromirrors, piezoresistive sensor, electrostatic-driven, laser beam scanning

## Abstract

The MEMS scanning micromirror requires angle sensors to provide real-time angle feedback during operation, ensuring a stable and accurate deflection of the micromirror. This paper proposes a method for integrating piezoresistive sensors on the torsion axis of electrostatic MEMS micromirrors to detect the deflection angle. The design uses a multi-layer bonding process to realize a vertical comb-driven structure. The device structure is designed as a double-layer structure, in which the top layer is the ground layer and integrates with piezoresistive sensor. This approach avoids crosstalk between the applied drive voltage and the piezoresistive sensor. This design also optimizes the sensor’s size, improving sensitivity. A MEMS two-dimensional (2D) scanning micromirror with a 1 mm mirror diameter was designed and fabricated. The test results indicated that, in a vacuum environment, the torsional resonance frequencies of the micromirror’s fast axis and slow axis were 17.68 kHz and 2.225 kHz, respectively. When driving voltages of 33 V and 40 V were applied to the fast axis and slow axis of the micromirror, the corresponding optical scanning angles were 55° and 45°, respectively. The piezoresistive sensor effectively detects the micromirror’s deflection state, and optimizing the sensor’s size achieved a sensitivity of 13.87 mV/V/°. The output voltage of the piezoresistive sensor shows a good linear relationship with the micromirror’s deflection angle, enabling closed-loop feedback control of the electrostatic MEMS micromirror.

## 1. Introduction

MEMS micromirrors (also known as “MEMS micro-scanners” or “MEMS scanning mirrors”) refer to optical MEMS devices that integrate micro-reflective mirrors with MEMS actuators, fabricated with optical MEMS technology. These micromirrors are widely used in optical scanning applications such as LiDAR [1,2,3], projection displays [4,5], augmented reality (AR)/virtual reality (VR) [6], and optical communication [7], where precise control over the laser beam’s scanning position and direction is crucial for the accurate measurement of the target object’s location, angle, and speed [8,9,10]. Therefore, the real-time testing of the MEMS micromirror’s deflection angle is critical. However, various actuation methods for micromirrors often exhibit hysteresis effects, and external environmental factors such as temperature and humidity can affect the micromirror’s torsional position [11]. These factors can significantly impair the scanning performance, leading to instability in its operational behavior. Simple open-loop control, using voltage or current driving signals, cannot ensure the accuracy of the deflection angle. To monitor the micromirror’s real-time movement, an angle sensor is needed to track its position and provide feedback, enabling closed-loop control [12]. This allows for the adjustment and control of the micromirror’s movement to improve the scanning performance [13].

At present, there are four sensing modes of MEMS micromirrors. Piezoresistive sensing utilizes the piezoresistive effect of single-crystal silicon and micro/nano fabrication techniques to create sensors. In 2016, Jan Grahmann et al. developed a new piezoresistive sensing technology for detecting the deflection of resonant and quasi-static micromirrors, enabled closed-loop phase-locked feedback control of the micromirror, improving the position resolution and trajectory accuracy of its movement [14]. Capacitive sensing measures position by detecting the capacitance between electrode plates. In 2015, Thomas et al. used an integrated vertical comb structure to synchronize the torsional movement of a 2D MEMS scanning micromirror oscillating in resonance [15]. In 2018, Xiamen University reported an integrated optoelectronic position sensor capable of measuring the linear displacement and tilt angle of a thermally actuated MEMS scanning mirror, facilitating the implementation of closed-loop feedback control [16]. Piezoelectric sensing utilizes the properties of piezoelectric materials to generate an electrical charge variation in response to mechanical changes. In 2007, Kobayashi et al. reported a piezoelectrically driven MEMS micromirror integrated with a piezoelectric torsional sensor. The micromirror surface was connected to the actuator through a hinge structure, and a PZT (lead zirconate titanate) thin film was deposited on the hinge, forming the piezoelectric torsional sensor [17].

In comparison, for electrostatically driven MEMS micromirrors, optical sensing requires the integration of photodetectors and other optical components, resulting in a complex structure, low integration density, and a relatively large overall size. Additionally, precise optical alignment is required, and the system must be shielded from interference caused by ambient light [18]. Piezoelectric sensing relies on the deposition of piezoelectric films, which are highly sensitive to temperature variations, increasing fabrication complexity and making this approach less suitable for electrostatic-driven MEMS micromirrors [19]. Additionally, the capacitive detection signals tend to be relatively weak, resulting in more complex readout circuits that are also susceptible to interference from the driving signals [20]. After a comprehensive comparison, this paper integrates piezoresistive sensors into the electrostatic MEMS micromirrors due to their small size and ease of integration. Piezoresistive sensors have already been successfully applied in electromagnetic-driven MEMS micromirrors. The sensor’s output voltage changes with stress variations at the torsional axis where it is integrated.

When the fast axis and slow axis of an electrostatic micromirror are driven to twist simultaneously, some structures of the micromirror are at a high drive voltage, which can affect the piezoresistive output voltage. For the piezoresistive sensors integrated with the micromirror’s fast axis and slow axis to achieve a stable output voltage, they need to be at the ground terminal. Therefore, we propose a new fabrication method for two-dimensional micromirrors. In this work, a multi-layer bonding process was utilized to create a vertical comb-driven structure, forming a two-layer device configuration. This method effectively resolves the crosstalk issue between the driving voltage and the piezoresistive sensor. A piezoresistive sensor was integrated into the electrostatic 2D MEMS scanning micromirror with vertical actuation, and by optimizing the size of the piezoresistive sensor, the sensitivity was increased to 13.87 mV/V/°.

This paper presents the development and testing of an electrostatic-driven 2D MEMS scanning micromirror with integrated piezoresistive sensors, optimized for high-resonant torsion performance in vacuum environments. The device achieves significant angular scanning improvements and demonstrates reliable closed-loop control capabilities. This capability ensures high-resolution and high-quality projections for laser projection displays. It also provides stability and reliability for optical communication devices. Additionally, it facilitates high-resolution imaging and detection in optical and medical imaging systems.

## 2. Design of the Structure for Electrostatic-Driven MEMS Micromirrors

### 2.1. Principle of Electrostatic Actuation

**Figure 1:**
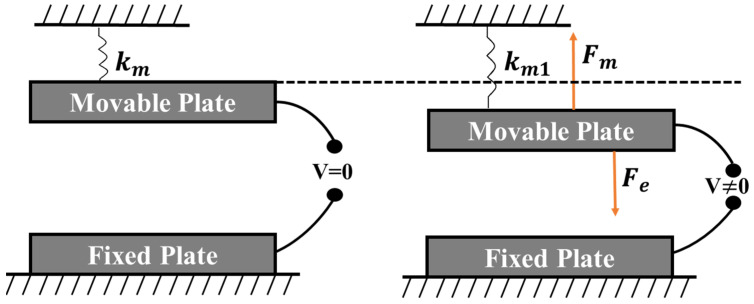
Schematic diagram of the parallel plate structure.

The driving structure of electrostatic MEMS micromirrors is based on a parallel plate configuration, which utilizes the electrostatic force between two electrode plates to induce deflection of the mirror surface [21]. As shown in Figure 1, the parallel plate structure mainly consists of two layers: a fixed lower plate and a movable upper plate, positioned in parallel. The lower plate is fixed to a substrate, while the upper plate is supported by mechanical springs which allows it to move freely, with the spring constant denoted as $k_{m}$. When no voltage is applied, the system remains in a static equilibrium, where both the displacement and mechanical restoring force are zero. When voltage V is applied, it generates an electrostatic force $F_{e}$, balanced by a mechanical restoring force, $F_{m}$, causing the movable electrode to undergo a certain displacement, with the spring constant denoted as $k_{m1}$. Due to the typically small mass of the plates, the effects of gravity do not produce significant static displacement; therefore, the influence of gravity is typically neglected in the static analysis of micro-scale MEMS devices. The parallel plate structure is primarily used for linear motion or rotational motion perpendicular to the electrodes.

In the case of a comb-driven structure, when the parallel plate capacitor formed by the movable and fixed comb fingers undergoes charging and discharging, electrostatic forces are generated in multiple directions. For comb-driven structures, when the parallel-plate capacitor formed by the movable and fixed comb fingers undergoes charging and discharging, the electric potential energy between a pair of comb electrodes is given by

(1) $$W=\frac{1}{2}CV^{2}=\frac{\varepsilon T_{c}Y_{o}}{\;2g}V^{2}$$

where C is the capacitance between the movable comb finger and the fixed comb finger, V is the driving voltage, ε is the permittivity of the air, $T_{c}$ represent the thickness of the comb finger, $Y_{o}$ represent the overlap length of movable and fixed comb finger, and g is the gap distance between the movable and fixed comb finger.

A single movable comb finger is affected by the fixed comb finger on the left and right sides. The total electric energy generated by comb fingers becomes

(2) $$W_{t}=NCV^{2}=\frac{N\varepsilon T_{c}Y_{o}}{\;g}V^{2}$$

where N is the number of comb finger.

**Figure 2:**
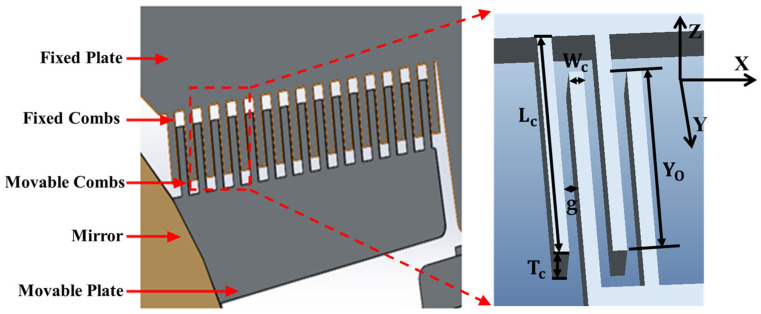
Schematic diagram of the comb-driven structure.

The deflection motion of the micromirror is primarily driven by the electrostatic force generated by the movable comb fingers along the Z direction. Figure 2 illustrates the structural model of a micromirror with an electrostatic comb-driven structure. A Cartesian coordinate system is established for the analysis, and using comb-driven structure as an example, in the X direction, a single movable comb finger experiences electrostatic forces in the X direction from the fixed comb fingers on both sides. In an ideal condition, where the gaps on both sides are equal, these forces cancel each other out. The magnitude of the driving force can be calculated as the derivative of the total stored energy with respect to the displacement in the direction of motion. Therefore, the electrostatic forces generated by the movable comb structure in the X, Y, and Z directions can be expressed as follows [22]:

(3) $$F_{x}=F_{left}-F_{right}=0;\;F_{y}=\frac{N\varepsilon T_{c}}{g}V^{2};\;F_{z}=\frac{N\varepsilon T_{c}Y_{o}}{g^{2}}V^{2}$$

### 2.2. Design of the Structure for Micromirrors

**Figure 3:**
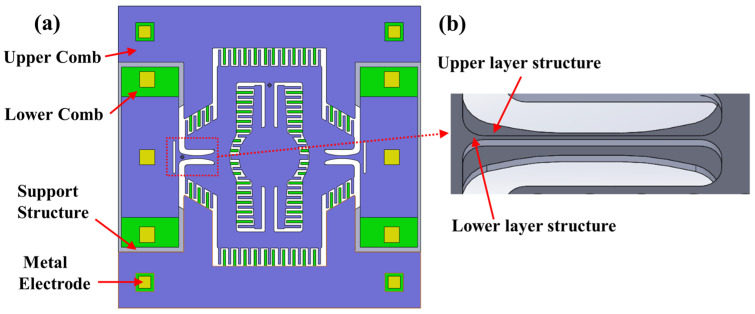
Schematic diagram of electrostatic-driven 2D MEMS micromirror structure. (**a**) The structure of the front side of the micromirror; (**b**) the structure of the slow axis double-layer ladder beam, presenting the structural view as seen from the back.

This paper presents the design of an electrostatic 2D MEMS scanning micromirror suitable for laser display applications. Compared to the horizontal comb structure, the vertical comb structure has a faster response, but the main design challenge is ensuring electrical insulation during simultaneous dual-axis operation. The structure of the micromirror driven by the vertical comb structure consists of five main components: a cavity support structure, a lower comb structure, an upper comb structure, a middle oxide layer, and a metal electrode structure. The detailed structural schematic is shown in Figure 3a. The support structure under the isolation groove first provides electrical insulation for the dual-axis drive of the micromirror, then serves as a connection for the upper device structure, and finally provides support for gold wire bonding after packaging. The slow axis structure utilizes a double-layer trapezoidal beam configuration, as illustrated in Figure 3b. The thinner structure of the lower axis applies driving voltage to the internal fast axis comb-driven structure, while also reducing the torsional resonance frequency of the slow axis, thereby increasing its torsional angle. The bottom cavity structure of the substrate not only provides space for the micromirror’s torsional movement but also protects the lower structures from damage during the fabrication process.

**Figure 4:**
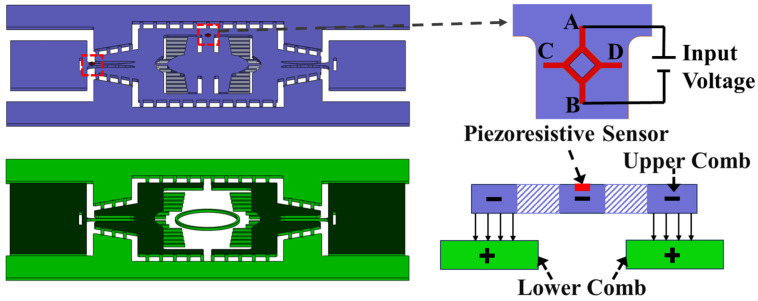
Schematic diagram of the integrated piezoresistive sensor position and working state potential.

The piezoresistive sensor enables the real-time detection of the micromirror’s deflection angle during operation [23,24,25]. As shown in Figure 4, the maximum stress is located at the midpoint of the long edge of the torsional axis; therefore, a four-terminal piezoresistive sensor is integrated at the end of the micromirror’s torsional axis. Typically, the sensor is formed by ion implantation on a silicon substrate, forming a doped layer, which alters the carrier mobility in single-crystal silicon. When the torsional axis of the micromirror twists, it experiences deformation that generates stress, causing the doped region to deform correspondingly and resulting in a change in resistivity. Under constant voltage applied between points A and B, any change in the sensor’s resistivity leads to a corresponding change in the output voltage measured between points C and D. The larger the torsional angle, the more pronounced the change in the output voltage. These voltage values serve as feedback signals by corresponding to different angles of rotation.

The device layer structure of the dual-axis electrostatic comb-driven MEMS micromirror consists of two main layers: the upper layer structure, which includes the movable comb structure and the mirror surface. The other one is the lower layer, which contains the fixed static comb structure. The position of the piezoresistive sensor is located above the torsional axis. When the driving voltage is applied, the entire upper layer structure of the micromirror is grounded to avoid crosstalk with the piezoresistive sensor. Figure 4 illustrates the specific loading of the driving potentials. The upper comb structure of the micromirror is maintained at zero potential, while the lower comb structure is connected to a high potential. This arrangement generates electrostatic forces between the comb fingers, allowing the micromirror to deflect in different directions. Here, the electrostatic forces generated by the comb fingers on the inner left and right sides of the micromirror enable resonant scanning around the fast axis, while the electrostatic forces generated by the comb fingers on the outer top and bottom sides allow resonant scanning around the slow axis.

To meet the resolution and refresh rate requirements in laser scanning display systems, we designed an electrostatic 2D MEMS scanning micromirror with a 1 mm mirror diameter. The initial design parameters for the micromirror are detailed in Table 1. After establishing the micromirror model according to the structural parameters, simulation results indicate that the torsional modal frequency of the slow axis is 1.976 kHz, while the torsional modal frequency of the fast axis is 18.297 kHz.

### 2.3. Design of the Piezoresistive Sensor

**Figure 5:**
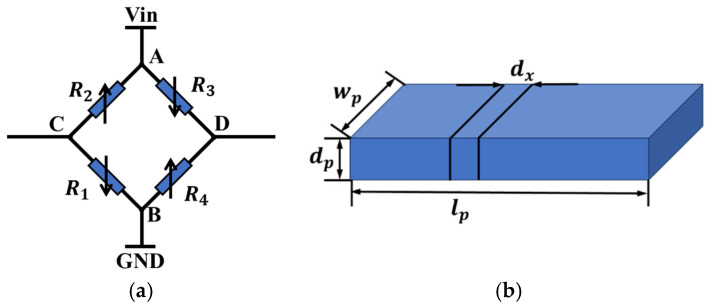
Schematic diagram of a piezoresistive sensor. (**a**) Wheatstone bridge circuit; (**b**) differential diagram of piezoresistive element.

The shape of the piezoresistive sensor adopts a Wheatstone bridge structure similar to that shown in Figure 5a. In this configuration, $R_{1}$, $R_{2}$, $R_{3}$, and $R_{4}$ are the four piezoresistive resistors in the Wheatstone bridge. the four resistors are symmetrically arranged in pairs. When the torsional axis is subjected to force and deforms, $R_{2}$ and $R_{4}$ exhibit a positive piezoresistive coefficient, resulting in an increase in resistance by ∆R, while $R_{1}$ and $R_{3}$ exhibit a relatively inverse variation coefficient, leading to a decrease in resistance by ∆R. The input voltage $V_{AB}$ is a DC bias voltage. After the resistance values change, the output signal $V_{CD}$ of the Wheatstone bridge (full bridge) can be expressed as

(4) $$V_{CD}=V_{AB}\frac{{(R}_{1}-∆R){(R}_{4}+∆R)-{(R}_{2}+∆R){(R}_{3}-∆R)}{{(R}_{1}-∆R{+R}_{4}+∆R){(R}_{2}+∆R{+R}_{3}-∆R)}$$

In an ideal state, the values of the four piezoresistive resistors are equal. When the change in resistance, ∆R, is much smaller than the resistance value, the output voltage can be approximated based on the linear relationship of the Wheatstone bridge. In this case, the output voltage $V_{CD}$ can be expressed as

(5) $$V_{CD}\approx V_{AB}\frac{∆R}{R_{1}}$$

In the equation, $R_{1}$, $R_{2}$, $R_{3}$, and $R_{4}$ represent the values of the four piezoresistive resistors, and ∆R is the change in piezo resistance.

The relative output sensitivity of the piezoresistive sensor can be expressed as

(6) $$S_{V}=\frac{V_{CD}}{V_{AB}}\approx \frac{∆R}{R_{1}}$$

The piezoresistive angle sensor operates based on the piezoresistive effect of single-crystal silicon. When the piezoresistive element undergoes external stress and deformation, the resistivity of the piezoresistor changes proportionally with the applied stress [26,27,28]. In this work, the piezoresistive angle sensor was fabricated using an N-type-doped single-crystal silicon. Through theoretical calculations, we determined that the piezoresistive unit has maximum sensitivity to torsional shear stress when oriented at odd multiples of 45° on (100) silicon wafer, making it suitable for angle detection in micromirrors [29,30,31]. After simplification, the expression for the piezoresistor’s resistance variation rate with respect to stress is given as follows:

(7) $$\frac{∆\rho }{\rho }=0.5\left(\sigma_{1}+\sigma_{2}\right)\left(\pi_{11}+\pi_{12}\right)+\sigma_{6}(\pi_{11}-\pi_{12})$$

where $\sigma_{1}$ and $\sigma_{2}$ represent the axial stress components in the piezoresistive effect, $\sigma_{6}$ denotes the shear stress component on the shear plane. $\pi_{11}$ and $\pi_{12}$ are the piezoresistive coefficients.

As shown in Figure 5b, a single resistor element in the sensor was considered for analysis. Assume that the thickness of the piezoresistive film is $d_{p}$, the width is $w_{p}$, and the length is $l_{p}$. The resistance change rate for a small element $d_{x}$ of the piezoresistor can be expressed as follows:

(8) $$\rho_{p}=\rho_{p0}+\rho_{p0}[0.5\left(\sigma_{1}+\sigma_{2}\right)\left(\pi_{11}+\pi_{12}\right)+\sigma_{6}\left(\pi_{11}-\pi_{12}\right)]$$

According to the resistivity formula, the resistance ${dR}_{p}$ of the microelement $d_{x}$ under stress is given by

(9) $${dR}_{p}=\rho_{p}\frac{d_{x}}{w_{p}d_{p}}=\rho_{p0}[1+0.5\left(\sigma_{1}+\sigma_{2}\right)\left(\pi_{11}+\pi_{12}\right)+\sigma_{6}\left(\pi_{11}-\pi_{12}\right)]\frac{d_{x}}{w_{p}d_{p}}$$

From the stress analysis on the torsional beam where the piezoresistor is located, it is known that the shear stress $\sigma_{6}\left(x\right)$ roughly follows a quadratic function along the length of the piezoresistor. Assuming $\sigma_{6}\left(x\right)=\sigma_{0}+Ax^{2}$ (where A<0) while neglecting changes in axial stress, the integral can be performed to derive the variation of the resistance $R_{p}$ with respect to the applied stress:

(10) $$R_{p}=\frac{\rho_{p0}[1+\left(\pi_{11}-\pi_{12}\right)\sigma_{0}]}{w_{p}d_{p}}l_{p}+\frac{\rho_{p0}A}{3w_{p}l_{p}}(\pi_{11}-\pi_{12}){l_{p}}^{3}$$

where $\sigma_{0}$ represents the stress constant, and $\rho_{p0}$ is the initial resistivity without applied stress, the initial resistance can be expressed as $R_{0}=\rho_{p0}\frac{l_{p}}{w_{p}d_{p}}$. Therefore, the change in the piezo resistor’s resistance under stress can be written as

(11) $$∆R=\frac{\rho_{p0}}{d_{p}}(\pi_{11}-\pi_{12})(\sigma_{0}\frac{l_{p}}{w_{p}}+\frac{A{l_{p}}^{3}}{3w_{p}})$$

Equation (9) shows that under the same shear stress, the larger the aspect ratio of the piezoresistive element, the greater the resistance change of a single resistor, which increases the output signal of the entire Wheatstone bridge, thus improving sensitivity.

**Figure 6:**
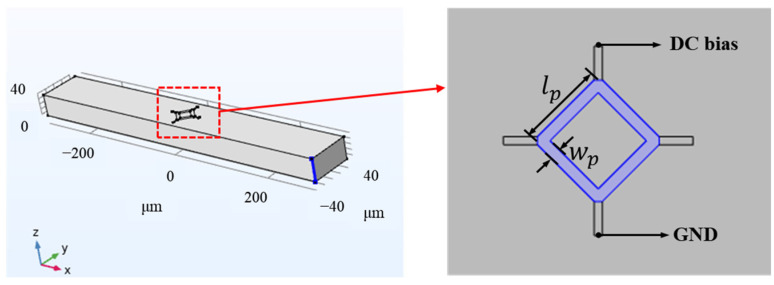
Model structure of a piezoresistive sensor.

This paper simulates the output voltages of piezoresistive sensors with different dimensions by establishing a model. The piezoresistive angle sensor is integrated into the micromirror’s torsion axis, utilizing the shear stress generated during torsion, inducing resistivity changes that lead to variations in the output voltage. As shown in Figure 6, the cantilever beam is used as the torsion model in the simulation, with a four-terminal piezoresistive sensor integrated onto the beam. The simulation couples two physical fields: solid mechanics and current in a single-layer shell. In the solid mechanics field, torque is applied to the cantilever beam to simulate the actual working conditions of the micromirror, generating shear stress in the piezoresistive region. In the current single-layer shell field, key properties of the piezoresistive material are defined, with the doping concentration set to $1.32\;\times \;10^{18}{\mathrm{cm}}^{-3}$, a doping depth of 600 nm, and a DC voltage of 3 V. These integrated physical processes enable accurate simulation and analysis of the piezoresistive sensor’s output voltage variations, providing performance evaluations and optimization pathways for practical applications.

**Figure 7:**
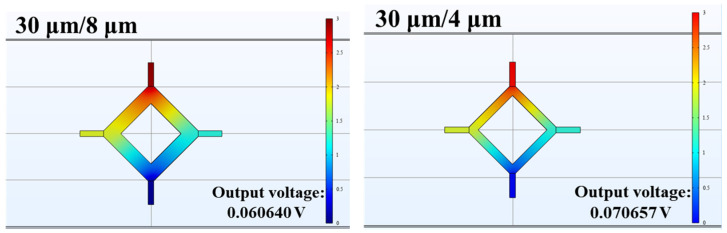
Simulation results of two different-sized piezoresistive sensors.

Here, we set the torsion angle of the torsion axis to be the same under the same load. At this point, the rotation angle of the axis was 28.4° and the shear stress in the piezoresistive region approximately 216.6 MPa, and we simulated the relative changes in the output signal amplitude of piezoresistive sensors with different aspect ratios. Under the same shear stress, the larger the aspect ratio of the piezoresistive element, the greater the change in resistance of a single resistor, resulting in a stronger output voltage from the entire Wheatstone bridge and consequently higher sensitivity. As shown in Figure 7, two different piezoresistive models with varying sizes were simulated. The lengths and widths of the piezoresistive sensors were set to 30 μm/8 μm and 30 μm/4 μm. The output voltages of the two piezoresistive sensors with different aspect ratios were obtained using COMSOL Multiphysics 6.1 software, as shown in Figure 7. The piezoresistive output voltages were 60.6 mV and 70.7 mV, respectively. The results indicate that the trend of output sensitivity of the piezoresistive sensor, as affected by its aspect ratio, is consistent with theoretical calculations.

## 3. Process Flow Design and Fabrication

**Figure 8:**
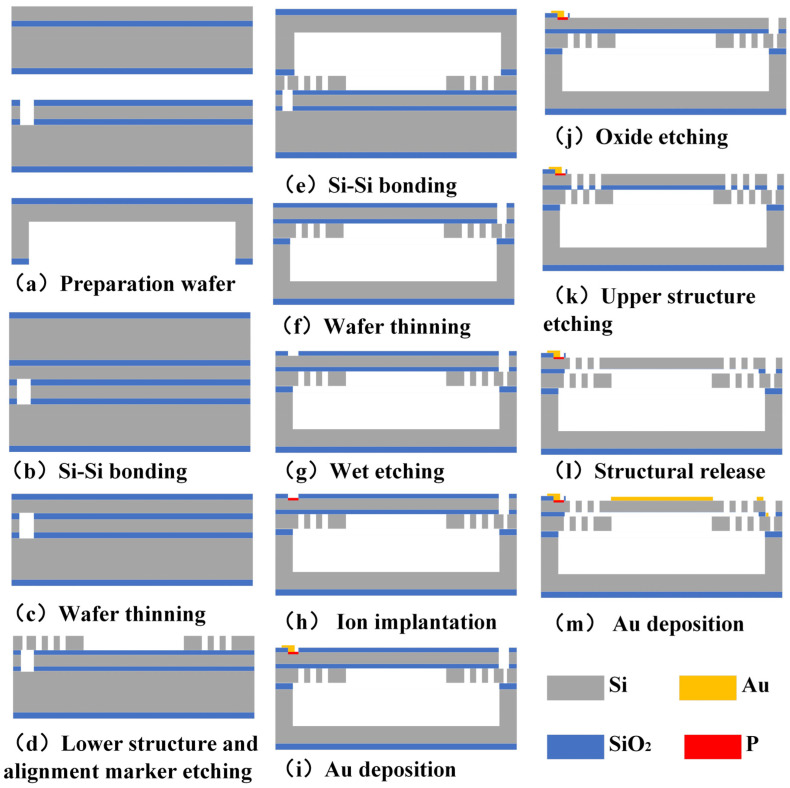
Fabrication process of the micromirror.

The challenge in fabricating the vertical comb-driven structure of electrostatic 2D MEMS scanning micromirror lies in the alignment precision between the upper and lower comb fingers. The fabrication process flow diagram for the mirror and the piezoresistive sensor is shown in Figure 8. In this study, we employ a multilayer bonding process to achieve a vertically stacked comb-driven structure of unequal heights. During the fabrication process, the lower comb structure and alignment marks are etched simultaneously. By utilizing the visibility of the window in the upper layer structure, the alignment between the upper and lower comb structures is achieved with only one stepper lithography alignment, reducing the number of alignment steps. This method enhances the alignment accuracy between the upper and lower comb finger structures.

The fabrication requires one silicon wafer for etching the cavity structure, along with two SOI wafers, each having a device layer thickness of 30 μm ± 1 μm. On the second SOI wafer, a large window is etched to facilitate alignment for the subsequent lithography of the upper comb structure. The window is positioned above the designed alignment marks and is larger than the area occupied by the marks. (Figure 8a). Steps (b) to (c) involve wafer bonding and thinning. The two prepared SOI wafers were bonded using silicon–silicon bonding, forming a double-layer SOI wafer structure. Thinning techniques were then employed to remove the substrate layer (Figure 8b,c). In step (d), the alignment marks required for stepper lithography, along with the lower comb structure, are etched onto the first layer of the wafer. This simultaneous etching process minimizes the need for additional alignment steps, thereby reducing alignment errors. The alignment marks were positioned directly above the window in the second layer structure and were used for alignment throughout the entire fabrication process (Figure 8d). Steps (e) and (f) represent the bonding to the device structure with the silicon wafer containing the cavity, forming the support and deflection space structure. Subsequently, the other half of the substrate layer was then removed to expose the alignment marker (Figure 8e,f). Steps (g) to (j) detail the fabrication process of the piezoresistive sensor, including the deposition and etching of the oxide layer, ion implantation of phosphorus ions, and magnetron sputtering of 30 nm Ti and 200 nm Au, followed by ion beam etching (IBE) etching to form the electrodes and leads (Figure 8g–j). Step (k) involves etching the upper comb structure onto the second layer of the wafer, using the alignment marks formed in step (d) for precise alignment. The presence of the window ensured that the alignment marks on the lower layer were fully visible, enabling accurate patterning and etching of the upper comb finger structure. This step also releases the intermediate silicon oxide layer (Figure 8k). The next step involves releasing the intermediate silicon oxide layer (Figure 8l). The final step involves ion beam evaporation of the electrode and mirror metal layers for reflecting the beam, ultimately resulting in the formation of the micromirror chip (Figure 8m).

The piezoresistive sensors were integrated at the ends of the torsional axes of the micromirror. Semiconductor doping was achieved through ion implantation into the single-crystal silicon device layer of the SOI wafer. The fabrication of the piezoresistive sensors involves several steps: wet etching of the oxide layer to form the piezoresistor pattern, ion implantation to create the doped regions, and high-temperature annealing to repair defects caused by ion implantation and activate the dopant ions. Finally, metal wiring is deposited and patterned through etching. Notably, the process does not involve deep silicon etching, thereby preserving the thickness of the device layer and ensuring the mechanical integrity of the micromirror.

**Figure 9:**
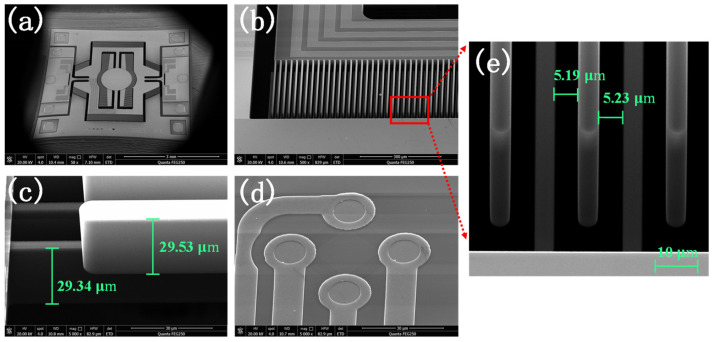
SEMs of a fabricated MEMS micromirror: (**a**) whole micromirror chip; (**b**) comb finger structure; (**c**) enlarged view of the vertical comb fingers (the data are the actual thickness); (**d**) four-terminal piezoresistive sensor; (**e**) comb fingers and gaps.

Figure 9 presents scanning electron microscope (SEM) images of the MEMS micromirror. Figure 9a shows an image of the whole micromirror chip fabricated using the process flow, including the mirror surface, torsion axis, comb finger structure, and piezoresistive sensor. The leads of the piezoresistive sensor for the fast axis are connected to the right edge through the slow axis linking the electrodes with the external packaging structure, while the leads of the slow axis piezoresistive sensor are directly connected at the left side. The bottom features a fixed support layer that can withstand significant stress during lead fabrication. Figure 9b illustrates the comb finger structure of the fabricated micromirror chip, and Figure 9c provides an enlarged view of the vertical comb fingers. It is evident that the comb fingers are etched cleanly, and the unequal-height comb finger structure is quite pronounced. The designed thickness of the comb fingers is 30 μm. Measurements show that the actual thickness of the movable comb finger is 29.53 μm, while the actual thickness of the fixed comb finger is 29.34 μm. Both measurements fall within the tolerance range specified for the SOI device layer thickness. Figure 9d displays the integrated four-terminal piezoresistive sensor, which has been fabricated successfully. Figure 9e shows the structure of the comb fingers and gaps. The measured gap on the right side of the movable comb finger is 5.23 µm, while the gap on the left side is 5.19 µm. Considering the effect of etching, the deviation is 0.23 µm.

## 4. Experiments and Discussion

**Figure 10:**
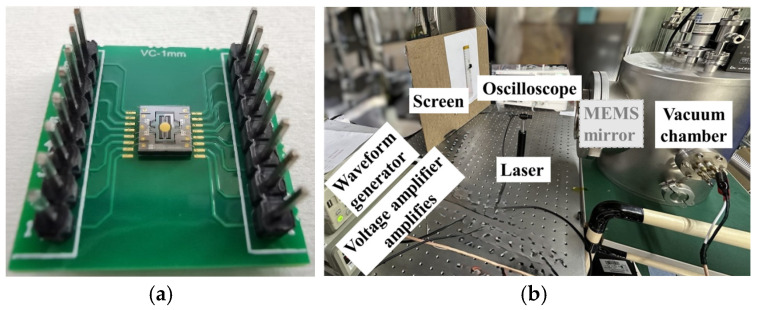
MEMS micromirror packaging and testing: (**a**) photos of an assemble MEMS micromirror; (**b**) actual experimental setup.

The electromechanical performance testing of the MEMS micromirror chip requires setting up a testing system. The West Bond wire bonder is utilized to connect the chip’s electrodes to the larger solder points on the external Printed Circuit Board (PCB), as shown in the final structure in Figure 10a. The wires on both sides facilitate connections to the driving signal source. As depicted in Figure 10b, during the mirror’s resonant torsion operation, the DG1022U dual-channel function/arbitrary waveform generator, produced by RIGOL Technologies, Inc. (Beijing, China), outputs the required waveform signals. A voltage amplifier then amplifies the peak-to-peak driving voltage. The selected laser is the HL63133DG red semiconductor laser source, produced by Ushio Company (Tokyo, Japan), with a wavelength of 638 nm. After collimation, the spot diameter is less than 1 mm. To achieve larger angles, the mirror was tested within a vacuum chamber, and an oscilloscope was used to detect the output signal from the piezoresistive sensors on the micromirror chip.

### 4.1. Testing the Scanning Angles of the Mirror in a Vacuum Environment

**Figure 11:**
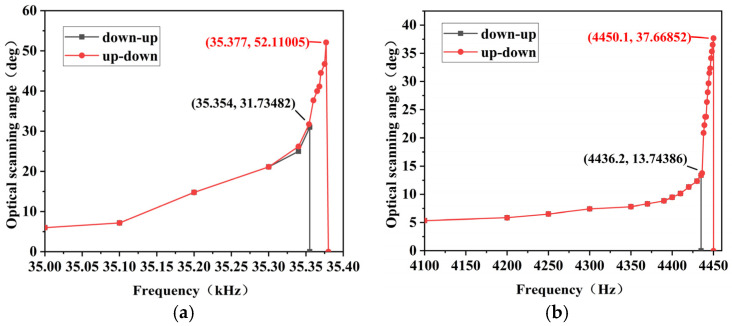
Results of the electrostatic MEMS micromirror frequency response: (**a**) frequency response curve of the fast axis; (**b**) frequency response curve of the slow axis.

After setting up the laser testing system, the packaged micromirror was positioned within the chamber, and the driving voltage was applied. A vacuum pump was used to reduce the pressure in the vacuum chamber to about 20 Pa, and the resonant twisting angles of the micromirror’s fast and slow axis were measured separately. Due to the use of a square wave signal with a 50% duty cycle to drive the micromirror, once the micromirror twists to a horizontal position and the driving force is removed, the torsion axis continues to induce deflection of the mirror surface in that direction due to its own inertia. Therefore, the torsional resonant frequency of the micromirror is half of the driving signal frequency. The results for the fast axis scanning are shown in Figure 11a. Under a 30 V square wave drive, the driving signal frequency was 35.377 kHz, resulting in an optical scanning full angle of 52.11°, with a resonant frequency of 17.68 kHz. The slow axis scanning results are illustrated in Figure 11b. With a 30 V square wave drive, the driving signal frequency was 4.450 kHz, leading to an optical scanning full angle of 37.66° and a resonant frequency of 2.225 kHz. The frequency response curves of the micromirror exhibited the characteristic frequency hysteresis behavior, typical of electrostatic micromirrors. The quasi-static test results of the micromirror chip show that at 100 V, the mechanical rotation angle of the micromirror’s fast axis is 0.12°, and that of the slow axis is 0.38°.

**Figure 12:**
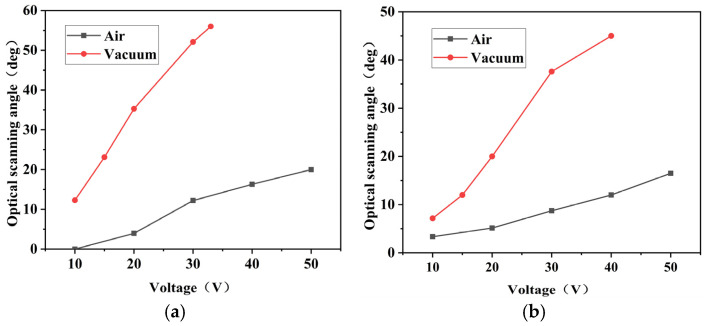
Results of the electrostatic MEMS micromirror amplitude–voltage characteristic: (**a**) amplitude–voltage characteristic of the fast axis; (**b**) amplitude–voltage characteristic of the slow axis.

After determining the twisting resonant frequencies of the micromirror’s fast and slow axis in a vacuum environment, the amplitude–voltage curves for the fast and slow axis were measured under both air and vacuum conditions. In the vacuum, the absence of air molecules leads to reduced damping effects, resulting in lower vibrational energy loss. Consequently, the twisting resonant frequency of the MEMS micromirror in a vacuum is generally slightly higher than its the designed resonant frequency. Figure 12a shows the scanning results of the micromirror around the fast axis. It can be observed that the mirror’s surface vibration starts with a driving voltage of 10 V, and the rotation angle gradually increases with increasing voltage. In the vacuum, the optical scanning full angle reaches 55° at a driving voltage of 33 V. Figure 12b illustrates the scanning results of the micromirror around the slow axis, and the optical scanning full angle of the micromirror’s surface reaches 45.2° at a driving voltage of 40 V. Comparative studies indicate that the rotation angle in the vacuum is 3.3 times larger than that in the air, significantly enhancing the resonant twisting angle of the MEMS micromirror and achieving the desired angular range.

### 4.2. Testing of the Piezoresistive Sensor

After testing the performance of the micromirror, the performance of the integrated piezoresistive sensor was also evaluated. Figure 10b shows an oscilloscope was used to receive the output signal from the piezoresistive sensor, with a supply voltage of 3 V applied to the four-terminal piezoresistive sensor.

**Figure 13:**
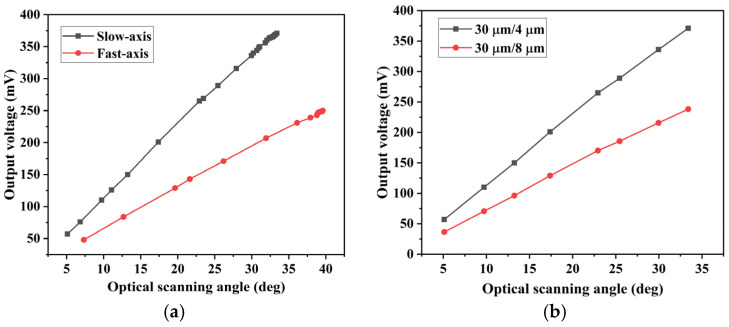
Results of the output characteristics of piezoresistive sensors: (**a**) output of piezoresistive sensors of different axes; (**b**) output of piezoresistive sensors of different sizes.

The output voltage characteristics of the sensor are depicted in Figure 13. In Figure 13a, the curve depicting the relationship curve between the output voltage amplitude of the piezoresistive sensor and the optical scanning angle of the micromirror shows a linear correlation. The different structures of the fast and slow axis of the micromirror result in varying shear stress during torsion, leading to a higher output voltage from the slow axis piezoresistive sensor compared to the fast axis sensor. When a driving voltage of 30 V is applied to the slow axis of the micromirror during resonant torsion, the optical scanning full angle of the mirror surface reaches 37.66°, with a mechanical rotation angle of 9.4°. At the maximum deflection angle, the output voltage is 392 mV, yielding a calculated sensitivity of 13.87 mV/V/°. To validate the effect of the aspect ratio of the piezoresistive unit on the output voltage, we incorporated two different aspect ratios during the fabrication process. By comparing the output voltages of the piezoresistive sensors, as shown in Figure 13b, it can be observed that the sensor with an aspect ratio of 30 μm/8 μm exhibited a sensitivity of 9.38 mV/V/°, while the sensor with an aspect ratio of 30 μm/4 μm demonstrated an output signal approximately 1.5 times higher under the same rotation angle. This demonstrates the influence of the sensor’s shape and size on its sensitivity, which corresponds well with the simulation and theoretical calculations. The excellent linearity of the piezoresistive sensor enables effective closed-loop feedback control of the micromirror’s torsion movement.

### 4.3. Testing of the 2D MEMS Scanning Micromirror

**Figure 14:**
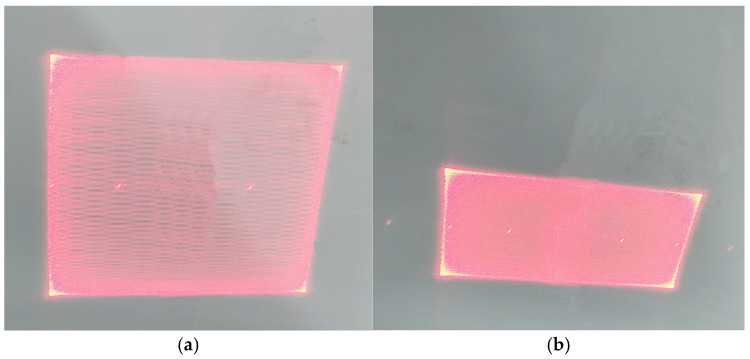
Image of micromirror dual-axis scanning: (**a**) image of Lissajous; (**b**) image of a raster scan.

The 2D scanning demonstration of the fabricated dual-axis vertical comb-driven MEMS micromirror was conducted. As shown in Figure 14, a square wave signal with a driving voltage of 30 V and frequencies of 35.337 kHz for the fast axis and 4.450 kHz for the slow axis was applied, generating a Lissajous scan pattern on the left. The fast axis drive was kept constant, while a sine wave signal with a frequency of 120 Hz was applied to the slow axis, producing the grating scan pattern on the right. The entire scanning process took place inside a vacuum chamber. Due to the vacuum chamber window being made of thick glass, the laser beam underwent multiple transmissions through the glass, leading to some blurriness in the scanned pattern.

## 5. Conclusions

Using micro-nano fabrication techniques, the piezoresistive sensor was integrated at the axis of an electrostatic 2D MEMS scanning micromirror, a vertically arranged comb-driven structure with varying heights was achieved through multilayer bonding, resulting in a micromirror with a diameter of 1 mm. This design positions the top layer of silicon in the device structure as the ground terminal, effectively avoiding interference from the driving electrical signals on the piezoresistive sensing. In comparison to air, the micromirror’s optical scanning full angle in a vacuum environment increased by 3.3 times. Experimental results indicated that the optical scanning full angle of the reflected light for the fast axis could reach 55°, while that for the slow axis could reach 45°. By optimizing the dimensions of the piezoresistive sensor, the sensitivity was improved to 13.87 mV/V/°. The excellent linearity of the piezoresistive sensor enables effective closed-loop feedback control of the micromirror’s torsion movement.

The dual-axis vertical comb-driven MEMS micromirror can achieve both Lissajous scanning and raster scanning. The electrostatic 2D MEMS scanning micromirror, integrated with a piezoresistive sensor, allows for precise control of the mirror’s surface position and orientation. This capability ensures high-resolution and high-quality projections for laser projection displays. It also provides stability and reliability for optical communication devices. Additionally, it facilitates high-resolution imaging and detection in optical and medical imaging systems.

## Figures and Tables

**Table 1:** Geometric parameters and material properties.

Symbol	Parameter	Value
D	Diameter of mirror	1 mm
$l_{1}$	Length of fast axis	800 μm
$W_{1}$	Width of fast axis	120 μm
$T_{1}$	Thickness of fast axis	30 μm
$l_{2}$	Length of slow axis	600 μm
$W_{2}$	Width of upper slow axis	50 μm
$W_{22}$	Width of lower slow axis	10 μm
$T_{2}$	Thickness of slow axis	60 μm
g	Gap between fixed and movable fingers	5 μm
$W_{c}$	Width of comb fingers	5 μm
$L_{c}$	Length of comb fingers	280 μm
$T_{c}$	Thickness of comb fingers	30 μm
$Y_{o}$	Length of the overlap of the comb finger	220 μm
$T_{o}$	Thickness of dielectric layer	1 μm
$N_{1}$	Number of comb fingers driving the fast axis	230
$N_{2}$	Number of comb fingers driving the slow axis	250

## Data Availability

The original contributions presented in the study are included in the article, further inquiries can be directed to the corresponding author.
